# Assessment of Rotator Cuff External Rotation: Isometric vs. Isotonic Testing Modes

**DOI:** 10.3390/jfmk11010029

**Published:** 2026-01-08

**Authors:** Luca Maestroni, Filippo Beretta, Fabio Civera, Paolo Artina, Marco Cuniberti, Francesco Bettariga, Anthony Turner

**Affiliations:** 1 ReAct, Via Madonna della Neve, 24, 24121 Bergamo, Italy; lucamae@hotmail.it (L.M.); fabio.civera@gmail.com (F.C.); pbd@hotmail.it (P.A.); 2 London Sport Institute, School of Science and Technology, Middlesex University, Greenlands Lane, London NW4 4BT, UK; 3 School of Medicine and Surgery, Università degli Studi Milano Bicocca, 20126 Milan, Italy; berettafilippo03@gmail.com; 4 School of Medicine and Health Science, Università degli Studi di Siena, 53100 Siena, Italy; marco.cuniberti@yahoo.it; 5 School of Sport and Health Sciences, University of Central Lancashire, Preston PR1 2HE, UK; 6 Exercise Medicine Research Institute, Edith Cowan University, Joondalup 6027, Australia; f.bettariga@ecu.edu.au; 7 School of Medical and Health Sciences, Edith Cowan University, Joondalup 6027, Australia

**Keywords:** rotator cuff, repetition maximum, assessment, performance

## Abstract

**Objectives**: To assess intra-session reliability of isometric and isotonic shoulder external rotation (ER) strength tests and to compare their outcomes. **Methods**: Thirty-eight healthy subjects (19 females; 19 males; 25.7 ± 6.0 years; 175 ± 9 cm; 70.3 ± 11.4 kg) completed a shoulder ER strength assessment including Prone and Standing ER Isometric tests and Seated 5 repetition maximum (RM) ER tests. Normality was checked with the Shapiro–Wilk test. Reliability was assessed using the CV and ICC (3, k, 95% CI). Linear mixed models examined sex and dominance effects. Correlations and multiple regression tested associations between tests (*p* < 0.05). **Results**: All tests performed displayed “excellent” reliability scores (CV from 1.9 to 3.1% and ICC from 0.970 to 0.994). No significant effect of dominance was observed in any strength test. Males showed significantly higher values than females in both Prone (3.8% higher, *p* < 0.001) and Standing (2.7% higher, *p* = 0.003) isometric ER strength tests. Prone and Standing isometric tests were moderately correlated (r = 0.62, 95% CI [0.46, 0.74], *p* < 0.001). A regression model explained 52.4% of the variance in Seated 5 RM ER strength (R^2^ = 0.524, *p* < 0.001), with Prone isometric strength emerging as a significant predictor (β = 0.612, *p* < 0.001). **Conclusions**: This study provides previously unreported 5 RM shoulder ER strength values in healthy adults, with all included tests showing excellent reliability. Isometric measures did not fully capture isotonic ER strength. Males outperformed females in isometric tests, but no gender difference was observed in Seated 5 RM strength.

## 1. Introduction

High-velocity overhead actions used in sports such as tennis, baseball, softball, American football, javelin, and volleyball share similar biomechanical demands, requiring refined technique to coordinate force generation across multiple body segments. Force is generated from the ground and transferred through the lower limbs and trunk to the upper limb, where the rotator cuff muscles assist in funneling forces through the glenohumeral joint and subsequently decelerating the throwing arm [1,2]. For example, in nationally ranked French tennis players, peak shoulder medial rotation velocity during fast serves reached approximately 1604 degrees/s at ball contact [3]. Weakness of the shoulder external rotator (ER) cuff muscles has been identified as a potential intrinsic risk factor for the development of shoulder injuries (i.e., rotator cuff and biceps tendinopathies, labral lesions, and acromioclavicular joint pain) in overhead athletes [4,5]. Furthermore, ER strength deficits have also been associated with shoulder pain in athletic and non-athletic populations [4,6,7,8,9,10,11,12]. Accurate quantification of shoulder ER strength is also essential for determining readiness to return-to-sport following shoulder stabilization procedures [13]. Healthcare and exercise professionals have adopted a variety of strength assessment modes and positions (i.e., manual muscle testing, force plates, isokinetic, and hand-held dynamometer [HHDs]) to determine the magnitude of shoulder strength [14,15,16,17,18]. Among these, isokinetic shoulder ER strength assessment can provide a reflection of rotator cuff muscular performance throughout the entire range of motion, including reports of rotational torque at angles of clinical interest (e.g., inner and outer ranges) [19]. HHDs have been demonstrated to be reliable and valid tools to measure shoulder strength at specific shoulder rotation angles [20,21], as well as the measurement of isometric strength, with the latter being highly correlated (r > 0.8) with isokinetic dynamometry testing [22,23]. Owing to cost and time restraints, isokinetic dynamometry may not be available to most clinicians; thus, HHDs could provide an appropriate alternative. Currently, their adoption among healthcare professionals remains limited [24,25], with more research required to validate this form of assessment.

Dynamic strength assessment using repetition maximum (RM) is widely employed to measure muscular performance across the entire range of motion in various muscle groups [26], including the shoulder (e.g., bench press). One-repetition maximum (1 RM) testing has demonstrated “good” to “excellent” reliability and is widely regarded as the gold standard for evaluating dynamic muscular strength [27]. However, 1 RM assessment is considered inappropriate for rotator cuff testing for two principal reasons: the smaller muscle groups involved compared to typical lower body assessments, and the need for familiarization due to the high load, which further limits its use in clinical settings. Therefore, for shoulder assessment, multiple RM tests (2–10 RM) are commonly used to estimate 1 RM, with good accuracy when the number of repetitions does not exceed 5 RM [28]. However, to our knowledge, multiple RM (>5 RM) are typically used to prescribe shoulder ER exercise intensity [29,30], yet their validity for accurately determining ER shoulder strength remains unexplored. Given that dumbbells are widely available in both sports and rehabilitation settings [29], this assessment mode could be easily implemented, provided its validity is established. Despite the widespread use of isotonic ER exercises in both rehabilitation and conditioning programs, the assessment of dynamic ER strength has not received sufficient attention compared to isometric or isokinetic assessment modes. A reliable, low-cost approach to assess ER strength across its entire available range could offer substantial practical value in rehabilitation and gym environments where access to specialized equipment is limited.

Different testing positions requiring isokinetic dynamometry or HHD have been used to measure ER isometric strength in non-athletic cohorts and in overhead athletes presenting with or without shoulder pain [6,9,14,15,19,31,32]. The Prone and Standing ER positions (elbow at 90° flexion, shoulder at 90° and 0° abduction, respectively) have been shown to preferentially recruit rotator cuff muscles [33], providing an accurate reflection of the rotator cuff maximal voluntary isometric capacity in ER [34]. Overall, ER cuff muscles exhibit a direction-specific activity during shoulder ER at 90° of abduction [35]. Additionally, when the arm is fully supported, axioscapular muscle activity is reduced [36]. Therefore, healthcare and exercise professionals may optimize the assessment of ER cuff muscles by selecting targeted shoulder positions and support conditions, thereby improving test specificity and enabling a detailed evaluation of shoulder muscle function.

To date, no studies have been conducted with the aim of assessing the reliability of isotonic shoulder ER strength testing using RM throughout the entire range of motion. Furthermore, it remains unclear whether this test modality can be considered interchangeable with isometric strength assessments. Therefore, further investigation into these assessment procedures is warranted to enhance understanding both in clinical and performance prospectives. Hence, this study aimed to (1) assess intra-session reliability scores of Prone and Standing isometric ER strength tests as well as Seated 5 RM ER strength test with the arm supported; (2) compare the outcomes of isometric and 5 RM strength testing procedures; and (3) report scores of each test for the dominant and non-dominant arm in a mixed-gender healthy population. To our knowledge, no prior studies have comprehensively evaluated these outcomes, representing a novel approach that may bridge the gap between training practices and clinical assessment needs.

## 2. Materials and Methods

### 2.1. Experimental Design

This cross-sectional observational study assessed intra-session reliability and examined the relationships between isometric and isotonic shoulder ER strength tests. All subjects were familiar with the test procedures and completed a standardized warm-up consisting of 1 min of aerobic training (SkiErg (Concept2, Morrisville, VT, USA)) at 60% of perceived maximal effort), followed by 10 external glenohumeral ER rotations with elbows kept close to the body, flexed at 90°, and wrists in a supinated position. Subsequently, 10 horizontal plane humeral abductions were performed, starting from a 90° flexed position with wrists also in supination. These warm-up exercises were executed using a 7–12 kg resistance band at an RPE of 2–3 (Lacertosus^®^ Rubber Power Band RED (mini), Parma, Italy). Finally, participants performed 10 Seated ER repetitions per shoulder of the isotonic test using a 1 kg weight [37]. All tests began with isometric assessments, performed first on the dominant arm and subsequently on the non-dominant arm. Arm dominance was determined as the preferred upper limb used by the subject to perform a ball-throwing task. The assessment was conducted under the supervision of an experienced investigator (>10 years using the stated test methodology).

### 2.2. Participants

A total of 38 subjects (19 females; 19 males; females: 24.6 ± 6.6 years, 168.5 ± 6.8 cm, 61.4 ± 7.6 kg; males: 26.1 ± 6.5 years, 180.2 ± 5.7 cm, 78.0 ± 7.3 kg [females: six volleyball, five gym, three swimming, two no regular sport participation, two climbing, one horse riding, one rugby, one triathlon, one CrossFit, and one athletic; males: five volleyball, three soccer, three tennis, three gym, two athletic, one basketball, one running, one triathlon, and one no regular sport participation]) volunteered to take part in this study. The data were collected during September 2025 at a private rehabilitation clinic in Bergamo (Italy). Recruitment of subjects was conducted through professional networks. Eligibility criteria for the study included the following: (1) age between 18 and 40 years, (2) full range of motion in internal and external shoulder rotation, and (3) absence of pain in the upper quadrant. Exclusion criteria were as follows: (1) presence of pain in the upper quadrant over the past three months (e.g., neck, thoracic, shoulder, wrist, and hand pain), (2) neurological disorders affecting upper limb function (e.g., stroke, peripheral neuropathy, and cervical radiculopathy), (3) previous shoulder surgery, and (4) discomfort affecting their ability to generate force during strength testing. This study was approved by the London Sport Institute Ethics Committee (ETH2526-0110).

### 2.3. Procedures

#### 2.3.1. Prone Isometric External Rotation Strength Test

**Figure 1:**
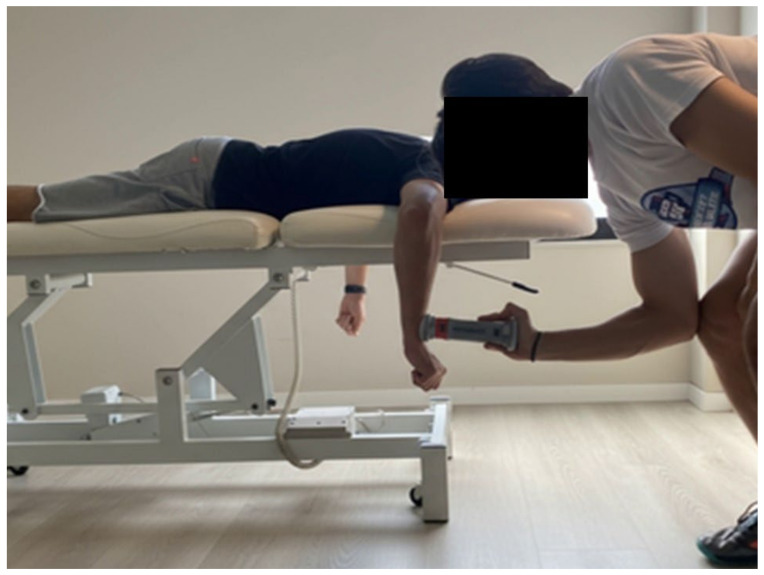
Prone isometric external rotation strength test.

The test was performed in the Prone position with the arm supported at 90° of abduction and neutral rotation. For each test, the subject was asked to perform glenohumeral ER against resistance applied by the HHD (DynaMo, VALD, Brisbane, Australia). A “make contraction” was used, and subjects were asked to gradually build their force to a maximum voluntary effort over a three-second period, then hold the maximal voluntary effort for five seconds. The examiner kept the HHD two centimeters proximal to the wrist joint line by matching the force exerted by the participant. Three measurements were collected for each performance, with a 30 s rest between tests, and the mean score was retained for statistical analysis. All subjects were verbally encouraged [6,18] (see Figure 1).

#### 2.3.2. Standing Isometric External Rotation Strength Test

**Figure 2:**
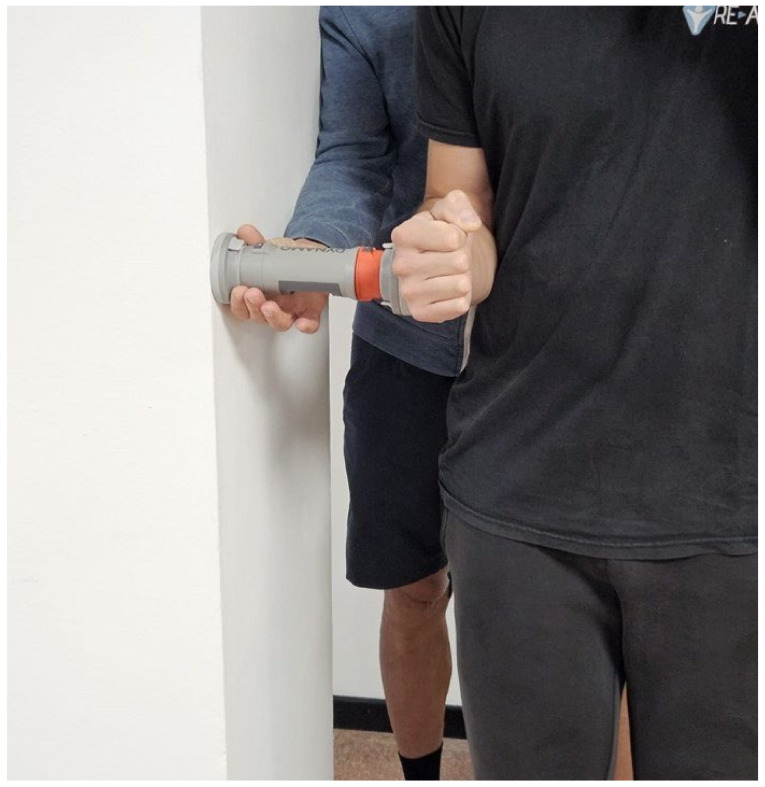
Standing isometric external rotation strength test.

The isometric testing was performed in a Standing position with the arm in a neutral position and the elbow flexed at 90°. For each test, the subject was asked to perform glenohumeral external rotation against resistance applied by the HHD (DynaMo, VALD, Brisbane, Australia), which was fixed against the wall. A “make contraction” was used, and subjects were asked to gradually build their force to a maximum voluntary effort over a three-second period, and then hold the maximal voluntary effort for five seconds. The examiner kept the HHD in place. The pressure (contact) point against the dynamometer, on the dorsal face of the forearm was applied two centimeters proximal to the wrist joint line. Three measurements were collected for each performance, with a 30 s rest between tests, and the mean score was retained for statistical analysis. All subjects were verbally encouraged (see Figure 2).

#### 2.3.3. Seated 5 RM External Rotation Strength Test

**Figure 3:**
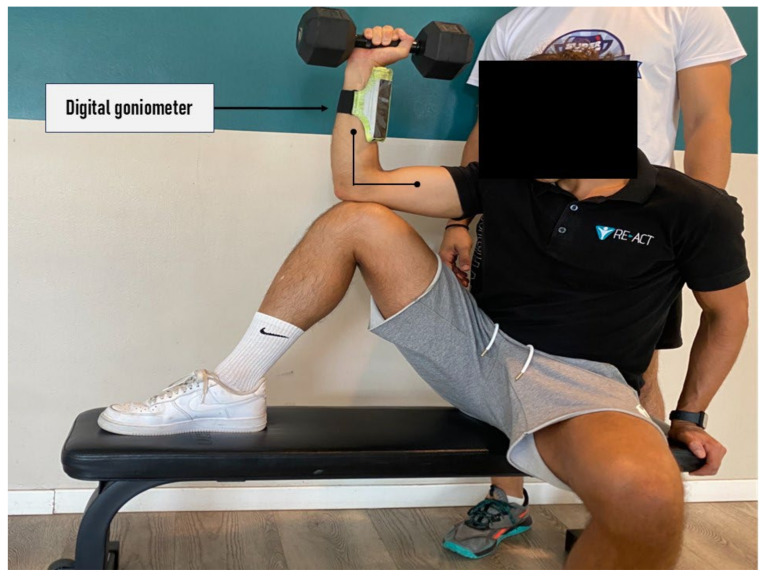
Seated 5 RM external rotation strength test.

The Seated 5 RM shoulder external rotation test was performed with the subject Seated comfortably on a flat bench using a dumbbell. One foot was placed on the bench with the knee bent, creating a stable base for the testing arm. The subject rested the elbow of the testing arm on the bent knee. The knee flexion was adjusted to maintain the shoulder at 90 degrees of abduction, and the elbow was kept at 90 degrees of flexion. The trunk was maintained in a frontal position. To monitor that subjects used the full available rotation ROM, an iPhone (Apple Inc., Cupertino, CA, USA) with a goniometer app was securely attached to the athlete’s forearm. To ensure the validity of each repetition, subjects were instructed to push as fast and hard as they could, achieving full excursion during each repetition (i.e., full external and internal rotation), while performing the movement in a controlled manner during the eccentric phase. The investigator dictated incremental load increases until 5 RM were achieved using the aforementioned technique within two to four attempts. Each series of repetitions was interspaced with 3–5 min of rest. Once 5 RM were achieved, a second 5 RM measurement was collected. The 5 RM values were then normalized to body weight, with the mean score retained for statistical analysis (see Figure 3).

#### 2.3.4. Statistical Analysis

The distribution of the data was checked using the Shapiro–Wilk normality test. Descriptive statistics (mean ± SD) for all variables were calculated. Intraclass correlation coefficients (ICC (3, k)) with their respective 95% confidence intervals (95% CIs), using a two-way mixed-effects model, were calculated. The coefficient of variation (CV) and its 95% CI were also calculated. Intra-session reliability scores were categorized as “acceptable” if the CV was ≤10% [38] and were further categorized as “excellent” if ICC was >0.90, “good” between 0.75 and 0.90, “moderate” between 0.50 and 0.75, and “poor” < 0.50 [39]. Linear mixed models were used to assess the effects of gender and dominance (fixed effects) on strength test scores. Limb was treated as a repeated measure to account for within-subject dependence. A multiple linear regression analysis was conducted to examine the relationship between Prone and Standing isometric ER strength test and the Seated 5 RM external rotation strength test. Assumptions for linear regression analysis—including multicollinearity (VIF), independence of residuals (Durbin–Watson), homoscedasticity, and normality of residuals—were tested and met. To determine the unique contribution of each independent variable after accounting for the other variables, partial correlations were calculated. The model was run using the “Enter” method, which entered all independent variables simultaneously. Partial correlation coefficients (r) were computed to assess the unique contribution of each predictor. The partial correlation for each predictor was interpreted as the proportion of variance in the dependent variable explained by that predictor, after controlling for the other predictors in the model. Correlation coefficient r were categorized as follows: 0.00–0.19 “very weak”, 0.20–0.39 “weak”, “0.40–0.59 “moderate, 0.60–0.79 “strong”, and 0.80–1.00 “very strong”. Unstandardized coefficients and R^2^ values were reported. The magnitude of the relationship based on the value of the coefficient of determination (r^2^) was interpreted using the following classification: r^2^ value of 0.02, 0.13, and 0.26 for small, moderate, and large effects, respectively [40]. Statistical significance was set at *p* < 0.05. All data were entered and organized in Microsoft Excel^®^ 2010, and all statistical analyses, including descriptive statistics, reliability (CV and ICC), linear mixed models, correlations, and regression analyses, were conducted using SPSS^®^ (v.25, Chicago, IL, USA). Similar to previous studies [18,23,31,41], a sample size of 19 subjects was required for a power of 95% and a significance level of 5%, for an expected ICC of 0.94 and a minimum acceptable ICC of 0.75 [42].

## 3. Results

Relative Prone and Standing isometric ER strength and Seated 5 RM ER strength scores of the dominant and non-dominant limbs, and their respective reliability scores, are reported in Table 1.

### 3.1. Dominance

No significant effect of limb dominance was observed in any of the strength tests performed.

### 3.2. Gender

For the Prone isometric ER strength test, there was a significant effect of gender (F(1, 36.005) = 12.832, *p* < 0.001), with a mean difference of 0.038 (3.8%) (SE = 0.010, 95% CI [0.016, 0.059], *p* < 0.001) in favor of males. For the Standing isometric ER strength test, there was a significant effect of gender (F(1, 36.000) = 10.051, *p* = 0.003), with a mean difference of 0.027 (2.7%) (SE = 0.009, 95% CI [0.010, 0.044], *p* = 0.003) in favor of males.

### 3.3. Correlation Between Isometric Tests

Prone and Standing isometric ER strength tests displayed a moderate significant correlation (r = 0.62, 95% CI [0.46, 0.74], *p* < 0.001). Correlations were similar across males and females and between dominant and non-dominant limbs, with no significant differences observed.

### 3.4. Regression Analysis

The overall regression model for Seated 5 RM ER strength was statistically significant (F(2, 73) = 40.134, *p* < 0.001, R^2^ = 0.524), indicating that the model explained 52.4% of the variance in Seated 5 RM ER strength. Prone isometric ER strength was a significant predictor of Seated 5 RM ER strength after controlling for the other predictor (β = 0.612, B = 0.436, *p* < 0.001, partial r = 0.57). In contrast, Standing isometric ER strength did not significantly contribute to the model (*p* = 0.122) when controlling for Prone isometric ER strength.

## 4. Discussion

All isometric and isotonic shoulder ER strength tests showed “excellent” intra-session reliability, with CV ranging from 1.9 to 3.1% and ICC from 0.970 to 0.994. Second, isometric ER strength assessed in Prone and Standing positions did not fully reflect isotonic ER strength, suggesting that these measures do not fully capture force production across the entire available range of motion. Finally, while males demonstrated greater relative isometric strength than females, no gender- or dominance-related differences were observed in Seated 5 RM ER strength. The consistently “excellent” reliability observed across all measures supports their appropriateness for both research and applied practice Our results for the isometric strength tests were similar to those reported in the literature [14,18], with Bettariga et al. [18] assessing healthy adult recreational athletes, and Cools et al. [14] providing reference values for overhead athletes, including competitive swimmers, volleyball players, and tennis players. Our isotonic ER strength assessment using the Seated 5 RM ER strength test revealed “excellent” reliability scores in line with current evidence on RM testing [26,27,28]. Clinically, given the availability of dumbbells in sports and rehabilitation settings, this test could be adopted to assess ER strength for injury prevention strategies or to monitor recovery of the ER cuff muscles during rehabilitation.

To our knowledge, this was the first study that compared isometric ER strength tests using HHDs and an isotonic ER strength test using dumbbells. The findings suggest that the Prone and Standing isometric ER strength measures do not comprehensively represent shoulder ER strength throughout the available range of motion. Therefore, reliance solely on isometric measures for evaluating shoulder ER strength in performance and rehabilitation contexts should be approached with caution. Indeed, the Prone and Standing Isometric ER strength tests predicted around half of the variance in the Seated 5 RM ER strength scores and were only moderately correlated with each other. This moderate correlation may be attributed to different levels of ER cuff activity at different abduction angles. Greater levels of activity of all portions of the infraspinatus and supraspinatus are recorded during ER exertions at greater abduction angles in comparison with the neutral arm position [34,43], which may explain why the Prone position, with the shoulder supported, better predicts dynamic ER strength than the Standing position. These biomechanical variations highlight why ER strength assessed at only one position may not accurately reflect performance in others. Consequently, these tests should not be considered interchangeable, and relying solely on isometric measurement of shoulder ER may lead to an overestimation of force production capacity across the available range of motion. This finding is of clinical relevance for practitioners dealing with overhead athletes and shoulder rehabilitation. Isometric assessments are widely used due to their tightly controlled application of force at specific joint angles, their ability to develop greater force than concentric contractions and their excellent reliability in assessing and tracking force production [44]. However, the findings of our study indicate that isometric testing performed at a single-joint angle does not completely correlate with results obtained from alternative isometric testing positions. Importantly, this approach alone is insufficient for a comprehensive assessment of shoulder function, as it is essential to consider multiple points throughout the range of motion (e.g., inner and outer ranges). This is analogous to isometric assessments performed with a stabilized dynamometer at 90°, 60°, and 30° of knee flexion from full extension in patients following anterior cruciate ligament reconstruction, which are necessary to fully characterize quadriceps strength [45]. The Seated 5 RM ER strength tests, as applied in this study to healthy individuals, may serve as a method to support injury prevention strategies and to evaluate full recovery in individuals with a history of shoulder disorders. Isometric assessments should be considered when pain interference across part of the available range of motion masks shoulder ER force generation capacity, or when longitudinal monitoring of force production at a specific joint angle is required. For example, at four and six months following glenohumeral joint stabilization surgery, male contact and collision athletes still exhibited significant isokinetic ER strength deficits at 90° of abduction [46]. These deficits were even more pronounced at the inner range of ER [46], suggesting that overall ER strength assessments could be complemented by targeted isometric testing to better detect residual deficits. Overall, multi-angle assessment approaches provide a more comprehensive evaluation of shoulder ER function, allowing clinicians to identify range-specific weaknesses that may be overlooked when relying on a single testing position.

Across the strength tests included in this study, no differences in arm dominance were found, in line with previous studies in healthy adults [15,47]. This aligns with our sample characteristics, as participants were not predominantly overhead athletes. In such population, greater values in the dominant arm can be expected [14]. Males showed greater relative values than females in isometric tests, but no difference in strength was reported in the Seated 5 RM strength test. Previous research has indicated strength levels varying significantly between men and women in Prone isometric ER strength [15], and thus achievable benchmarks may wish to reflect gender differences. Our data were collected in a relatively young, healthy population, with scores close to those of similar cohorts and consistent with reference values reported for recreational and overhead athletes, including swimmers, volleyball players, and tennis players [15,18]. Future normative datasets may benefit from stratifying values by gender, training background, and primary sport.

Our data were limited to relatively young, healthy adults, and no subjects with shoulder injuries or clinical pathologies were included. Therefore, generalization of these results to clinical populations requires caution, and further studies assessing shoulder ER force generation capacity using RM across different shoulder pathologies and recovery stages are needed. The tests included in this study were limited to movements involving ER actions. We did not consider internal rotation and multi-joint strength tests (e.g., bench press), which are important characteristics for intra-limb symmetry and global upper limb force generation capacity and could thus be considered for future studies. Additionally, our subjects practiced a range of sports, not all of which impose substantial demands on the shoulder, introducing potential variability in rotator cuff force production that should be considered when interpreting these findings. Finally, only intra-session reliability was assessed, and inter-session reliability remains untested.

## 5. Conclusions

The findings of the current study provide previously unreported relative dynamic shoulder ER strength scores using 5 RM in healthy adults. All isometric and isotonic tests performed displayed “excellent” reliability scores. The Prone and Standing isometric ER strength measures do not fully reflect shoulder ER strength throughout the available range of motion. Males showed greater relative values than females in isometric tests, but no difference was found in the Seated 5 RM strength test. No difference was found according to arm dominance. Future studies should examine these tests in clinical populations and define return-to-sport benchmarks.

## Figures and Tables

**Table 1:** Isometric and isotonic external rotation (ER) strength values, along with their respective reliability scores. This includes mean, standard deviation, and 95% confidence intervals (CIs).

Test			CV (95% CI) Dominant Limb	ICC (95% CI) Dominant Limb	CV (95% CI) Non-Dominant Limb	ICC (95% CI) Non-Dominant Limb
	Dominant Limb (95% CI)	Non-Dominant Limb (95% CI)				
Prone ER Isometric Test (kg/kg)	0.24 ± 0.04 [0.23, 0.26]	0.24 ± 0.04 [0.22, 0.25]	2.9 (2.4–3.5)	0.987 (0.978–0.993)	2.7 (2.2–3.2)	0.987 (0.978–0.993)
Standing ER Isometric Test (kg/kg)	0.21 ± 0.03 [0.20, 0.22]	0.21 ± 0.03 [0.20, 0.22]	3.1 (2.7–3.6)	0.983 (0.970–0.991)	2.9 (2.5–3.3)	0.984 (0.972–0.992)
Seated 5 RM ER test (kg/kg)	0.10 ± 0.03 [0.09, 0.11]	0.10 ± 0.03 [0.09, 0.11]	1.9 (1.4–2.3)	0.997 (0.994–0.998)	1.9 (1.0–2.8)	0.994 (0.989–0.997)

Values are reported relative to body mass (kg/kg), RM (repetition maximum).

## Data Availability

The data presented in this study are available on request from the corresponding author. The data are not publicly available due to privacy or ethical restrictions.

## Abbreviations

The following abbreviations are used in this manuscript:

ER	External rotators
HHD	Hand-held dynamometer
RM	Repetition maximum

## References

[1] Chu S.K., Jayabalan P., Kibler W.B., Press J. (2016). The kinetic chain revisited: New concepts on throwing mechanics and injury. PM&R.

[2] Seroyer S.T., Nho S.J., Bach B.R., Bush-Joseph C.A., Nicholson G.P., Romeo A.A. (2010). The kinetic chain in overhand pitching: Its potential role for performance enhancement and injury prevention. Sports Health.

[3] Gorce P., Jacquier-Bret J. (2024). Are there kinematic and kinetic parameters correlated with racket velocity during the tennis serve? A preliminary comparison between a slow and a fast serve for performance improvement. Front. Sports Act. Living.

[4] Byram I.R., Bushnell B.D., Dugger K., Charron K., Harrell F.E., Noonan T.J. (2010). Preseason shoulder strength measurements in professional baseball pitchers: Identifying players at risk for injury. Am. J. Sports Med..

[5] Kwan C.-K., Ko M.-C., Fu S.-C., Leong H.-T., Ling S.K.-K., Oh J.-H., Yung P.S.-H. (2021). Are muscle weakness and stiffness risk factors of the development of rotator cuff tendinopathy in overhead athletes: A systematic review. Ther. Adv. Chronic Dis..

[6] Maestroni L., Marelli M., Gritti M., Civera F., Rabey M. (2020). External rotator strength deficits in non-athletic people with rotator cuff related shoulder pain are not associated with pain intensity or disability levels. Musculoskelet. Sci. Pract..

[7] Vigolvino L.P., Barros B.R.S., Medeiros C.E.B., Pinheiro S.M., Sousa C.O. (2020). Analysis of the presence and influence of Glenohumeral Internal Rotation Deficit on posterior stiffness and isometric shoulder rotators strength ratio in recreational and amateur handball players. Phys. Ther. Sport.

[8] Miller J.E., Higgins L.D., Dong Y., Collins J.E., Bean J.F., Seitz A.L., Katz J.N., Jain N.B. (2016). Association of Strength Measurement with Rotator Cuff Tear in Patients with Shoulder Pain: The Rotator Cuff Outcomes Workgroup Study. Am. J. Phys. Med. Rehabil..

[9] McLaine S.J., Ginn K.A., Fell J.W., Bird M.-L. (2018). Isometric shoulder strength in young swimmers. J. Sci. Med. Sport.

[10] Lee S.M., Seo Y.G., Park W.H., Yoo J.C. (2020). Preoperative Rotator Muscle Strength Ratio Predicts Shoulder Function in Patients After Rotator Cuff Repair. Orthop. J. Sports Med..

[11] McLaine S.J., Bird M.-L., Ginn K.A., Hartley T., Fell J.W. (2019). Shoulder extension strength: A potential risk factor for shoulder pain in young swimmers?. J. Sci. Med. Sport.

[12] Hams A.H., Evans K., Adams R., Waddington G., Witchalls J. (2019). Shoulder internal and external rotation strength and prediction of subsequent injury in water-polo players. Scand. J. Med. Sci. Sports.

[13] Myers H., Wulff K., Antonelli C., Bokshan S., Hendren S., Lau B.C. (2024). Objective measures for assessing readiness to return to sport after shoulder instability procedures are not standardized: A systematic review. Arthrosc. Sports Med. Rehabil..

[14] Cools A.M., Vanderstukken F., Vereecken F., Duprez M., Heyman K., Goethals N., Johansson F. (2016). Eccentric and isometric shoulder rotator cuff strength testing using a hand-held dynamometer: Reference values for overhead athletes. Knee Surg. Sports Traumatol. Arthrosc..

[15] Riemann B.L., Davies G.J., Ludwig L., Gardenhour H. (2010). Hand-held dynamometer testing of the internal and external rotator musculature based on selected positions to establish normative data and unilateral ratios. J. Shoulder Elb. Surg..

[16] Nagatomi T., Mae T., Nagafuchi T., Yamada S.I., Nagai K., Yoneda M. (2017). Shoulder manual muscle resistance test cannot fully detect muscle weakness. Knee Surg. Sports Traumatol. Arthrosc..

[17] Ashworth B., Hogben P., Singh N., Tulloch L., Cohen D.D. (2018). The Athletic Shoulder (ASH) test: Reliability of a novel upper body isometric strength test in elite rugby players. BMJ Open Sport Exerc. Med..

[18] Bettariga F., Lopomo N.F., Civera F., Lazzarini S.G., Mantovani L., Maestroni L. (2024). Reliability and validity of hand-held dynamometer and hand-held sphygmomanometer for testing shoulder isometric external and internal rotator muscles strength. J. Sci. Sport Exerc..

[19] Fanning E., Falvey E., Daniels K., Cools A. (2022). Angle-specific isokinetic shoulder rotational strength can be reliably assessed in collision and contact athletes. J. Sport Rehabil..

[20] Stark T., Walker B., Phillips J.K., Fejer R., Beck R. (2011). Hand-held dynamometry correlation with the gold standard isokinetic dynamometry: A systematic review. PM&R.

[21] Dollings H., Sandford F., O’ Conaire E., Lewis J. (2017). Shoulder Strength Testing: The Intra- and Inter-Tester Reliability of Routine Clinical Tests, Using the PowerTrack™ II Commander. Shoulder Elb..

[22] Roy J.S., MacDermid J.C., Orton B., Tran T., Faber K.J., Drosdowech D., Athwal G.S. (2009). The concurrent validity of a hand-held versus a stationary dynamometer in testing isometric shoulder strength. J. Hand Ther..

[23] Holt K.L., Raper D.P., Boettcher C.E., Waddington G.S., Drew M.K. (2016). Hand-held dynamometry strength measures for internal and external rotation demonstrate superior reliability, lower minimal detectable change and higher correlation to isokinetic dynamometry than externally-fixed dynamometry of the shoulder. Phys. Ther. Sport.

[24] Aguilaniu A., Delvaux F., Schwartz C., Martens G., Forthomme B., Kaux J.F., Croisier J.L. (2023). Survey of physicians’ and physiotherapists’ ankle muscle strength assessment practices for safe return to sports after lateral ankle sprain: A short report. Physiother. Res. Int..

[25] Braun T., Rieckmann A., Weber F., Grüneberg C. (2018). Current use of measurement instruments by physiotherapists working in Germany: A cross-sectional online survey. BMC Health Serv. Res..

[26] Liguori G., American College of Sports Medicine (2020). ACSM’s Guidelines for Exercise Testing and Prescription.

[27] Grgic J., Lazinica B., Schoenfeld B.J., Pedisic Z. (2020). Test–retest reliability of the one-repetition maximum (1RM) strength assessment: A systematic review. Sports Med. Open.

[28] Reynolds J.M., Gordon T.J., Robergs R.A. (2006). Prediction of one repetition maximum strength from multiple repetition maximum testing and anthropometry. J. Strength. Cond. Res..

[29] Littlewood C., Malliaras P., Chance-Larsen K. (2015). Therapeutic exercise for rotator cuff tendinopathy: A systematic review of contextual factors and prescription parameters. Int. J. Rehabil. Res..

[30] Naunton J., Street G., Littlewood C., Haines T., Malliaras P. (2020). Effectiveness of progressive and resisted and non-progressive or non-resisted exercise in rotator cuff related shoulder pain: A systematic review and meta-analysis of randomized controlled trials. Clin. Rehabil..

[31] McLaine S.J., Ginn K.A., Kitic C.M., Fell J.W., Bird M.-L. (2016). The Reliability of Strength Tests Performed In Elevated Shoulder Positions Using a Handheld Dynamometer. J. Sport Rehabil..

[32] Leahy I., Florkiewicz E., Shotwell M.P. (2024). Isokinetic Dynamometry for External and Internal Rotation Shoulder Strength in Youth Athletes: A Scoping Review. Int. J. Sports Phys. Ther..

[33] Boettcher C.E., Ginn K.A., Cathers I. (2009). Which is the optimal exercise to strengthen supraspinatus?. Med. Sci. Sports Exerc..

[34] Edwards P.K., Ebert J.R., Littlewood C., Ackland T., Wang A. (2017). A systematic review of electromyography studies in normal shoulders to inform postoperative rehabilitation following rotator cuff repair. J. Orthop. Sports Phys. Ther..

[35] Boettcher C.E., Cathers I., Ginn K.A. (2010). The role of shoulder muscles is task specific. J. Sci. Med. Sport.

[36] Tardo D.T., Halaki M., Cathers I., Ginn K.A. (2013). Rotator cuff muscles perform different functional roles during shoulder external rotation exercises. Clin. Anat..

[37] McCrary J.M., Ackermann B.J., Halaki M. (2015). A systematic review of the effects of upper body warm-up on performance and injury. Br. J. Sports Med..

[38] Turner A., Brazier J., Bishop C., Chavda S., Cree J., Read P. (2015). Data Analysis for Strength and Conditioning Coaches: Using Excel to Analyze Reliability, Differences, and Relationships. Strength. Cond. J..

[39] Koo T.K., Li M.Y. (2016). A Guideline of Selecting and Reporting Intraclass Correlation Coefficients for Reliability Research. J. Chiropr. Med..

[40] Cohen J. (2013). Statistical Power Analysis for the Behavioral Sciences.

[41] Toohey L.A., Noronha M.d., Nunes G.S. (2017). The use of a sphygmomanometer to measure shoulder isometric strength: A validity and reliability study. Fisioter. Mov..

[42] Borg D.N., Bach A.J., O’Brien J.L., Sainani K.L. (2022). Calculating sample size for reliability studies. PM&R.

[43] Whittaker R.L., Alenabi T., Kim S.Y., Dickerson C.R. (2022). Regional electromyography of the infraspinatus and supraspinatus muscles during standing isometric external rotation exercises. Sports Health.

[44] Oranchuk D.J., Storey A.G., Nelson A.R., Cronin J.B. (2018). Isometric training and long-term adaptations; effects of muscle length, intensity and intent: A systematic review. Scand. J. Med. Sci. Sports.

[45] Beere M., Ebert J.R., Joss B., Ackland T. (2022). Isometric dynamometry, dependent on knee angle, is a suitable alternative to isokinetic dynamometry when evaluating quadriceps strength symmetry in patients following anterior cruciate ligament reconstruction. Knee.

[46] Fanning E., Daniels K.A., Cools A., Mullett H., Delaney R., Mcfadden C., Falvey E. (2024). Upper limb strength and performance deficits after glenohumeral joint stabilisation surgery in contact and collision athletes. Med. Sci. Sports Exerc..

[47] Bradley H., Pierpoint L. (2023). Normative values of isometric shoulder strength among healthy adults. Int. J. Sports Phys. Ther..

